# Radioablação Cardíaca não Invasiva para Doença de Chagas

**DOI:** 10.36660/abc.20230055

**Published:** 2023-03-16

**Authors:** Philip C. Wiener, Kaitlin M.S. Moore, Carlos Contreras, Clifford G. Robinson, Phillip S. Cuculich

**Affiliations:** 1 Washington University School of Medicine St. Louis MO EUA Washington University School of Medicine, St. Louis, MO – EUA

**Keywords:** Cardiomiopatia Chagásica, Doença de Chagas, Radiocirurgia/métodos, Usos de Radiação na Medicina e Pesquisa, Taquicardia Ventricular

A cardiopatia chagásica é uma causa comum de cardiomiopatia não isquêmica na América Latina.^1^ É causada pelo *Trypanosoma cruzi*, um parasita que causa miopericardite aguda e uma cardiomiopatia fibrosante mais crônica. Na maioria dos estudos, a causa geral mais comum de óbito é a parada cardíaca súbita por arritmias (55–60%), seguida por insuficiência cardíaca (25–30%) e eventos embólicos (10–15%). O uso de cardioversor-desfibrilador implantável (CDI) pode salvar vidas, em casos de arritmias ventriculares, mas é doloroso e está associado a padrões acelerados de insuficiência cardíaca e óbitos. A ablação por cateter pode ser uma terapia paliativa eficaz para muitos pacientes, a fim de minimizar os choques do CDI devido às taquicardias ventriculares (TV). Devido à natureza irregular transmural da cicatriz associada à doença de Chagas, os melhores resultados são obtidos com o mapeamento e ablação da cicatriz das superfícies endocárdica e epicárdica na tentativa de homogeneizar a cicatriz.^2^ E, no entanto, para muitos pacientes, a TV é recorrente e, com ela, surgem mais choques provenientes do CDI. As recorrências de TV podem resultar de circuitos tratados de forma incompleta na cicatriz ou da formação de novos circuitos ao longo do tempo.

Scanavacca et al.,^3^ relatam o primeiro uso de um tratamento totalmente não invasivo por meio de radiação focalizada em paciente portador da doença de Chagas para minimizar e, em seguida, eliminar a TV. O tratamento, conhecido como radiocirurgia estereotáxica corpórea (*Stereotactic Body Radioation Therapy* — SBRT), revolucionou a maneira como muitos pacientes com câncer são tratados. A SBRT é uma modalidade de radiação ablativa que emprega tecnologias de imagem avançadas capazes de danificar com precisão um tecido-alvo com altas doses de radiação, poupando os tecidos normais próximos. A SBRT cardíaca, ou radioablação cardíaca, requer estreita colaboração entre as equipes de ritmo cardíaco, oncologia de radiação e física médica para desenvolver planos de tratamento precisos e seguros em partes do miocárdio cicatrizado, evitando estruturas saudáveis próximas. Recentemente, esta tecnologia tem sido utilizada para tratar pacientes com TV recorrente com resultados promissores em pacientes com cardiomiopatias isquêmicas e diversas formas não isquêmicas.^4,5^

Então, por que Chagas? De muitas maneiras, a doença de Chagas pode ser uma cardiomiopatia ideal para fazer uso de radioablação cardíaca não invasiva. Primeiro, os circuitos de TV na doença de Chagas geralmente atravessam as camadas do miocárdio, usando canais de cicatriz epicárdica, miocárdica e endocárdica. Uma vantagem importante da radioablação cardíaca sobre o tratamento por cateter é que a radioablação é aplicada em toda a espessura do miocárdio cicatrizado. Em segundo lugar, a radioablação é um procedimento muito mais fácil de ser tolerado pelos pacientes. Procedimentos por cateter para pacientes com doença de Chagas, usando ablação endocárdica e epicárdica, podem ser demorados e arriscados. De fato, o paciente descrito no relato de caso sofreu diversas complicações conhecidas do mapeamento epicárdico, incluindo hemopericárdio e hemoperitônio. Com a radioablação cardíaca, o tratamento foi concluído em 15 minutos, sem a introdução de nenhum cateter no organismo. Em terceiro lugar, embora as arritmias ventriculares possam surgir de vários locais, a localização da cicatriz topográfica mais comum é a região inferolateral ventricular esquerda. Essa localização é adequada para a radioablação cardíaca. As estruturas luminais do sistema gastrointestinal (esôfago, estômago, intestino) são radiossensíveis, e raros efeitos adversos da radiação a esses órgãos foram descritos em pacientes submetidos à radioablação cardíaca.^6^

Os autores devem ser elogiados pelos diversos aspectos de cuidado e ponderação nesse caso. Primeiro, eles elegeram um paciente ideal para radioablação cardíaca, com mais sintomas relacionados à TV do que à insuficiência cardíaca. Para pacientes com sintomas de insuficiência cardíaca classe 4 da NYHA, é improvável que o tratamento bem-sucedido da TV com cateteres ou radiação afete a curta duração de sobrevida. Além disso, pacientes com cardiomiopatia avançada e níveis extremos de cicatrização miocárdica provavelmente terão muitas arritmias ventriculares que provavelmente não serão totalmente tratadas com um único procedimento. Nesse relato de caso, no entanto, os autores foram criteriosos em sua seleção de pacientes, escolhendo um paciente com sintomas leves a moderados de insuficiência cardíaca e que já havia tentado terapias convencionais. Se sua TV fosse controlada com radioablação, era esperado que ele continuasse em uma trajetória de vida razoável. Felizmente, essa foi sua experiência após a radioablação.

Em segundo lugar, de uma perspectiva técnica e cognitiva, a parte mais desafiadora da radioablação cardíaca para um eletrofisiologista é decidir o local a ser irradiado. Esse processo, denominado “*targeting*”, requer níveis avançados de conhecimento da anatomia cardíaca e interpretação de dados eletrofisiológicos, como mapeamentos feitos com cateteres e ECG de 12 derivações durante a TV. Os autores fizeram uso de diversas modalidades de imagem para localizar a cicatriz, incluindo mapeamentos anteriores de voltagem por cateter e tomografia computadorizada cardíaca. Em seguida, adicionaram cuidadosamente dados eletrofisiológicos provenientes de mapeamento de ativação por cateter e ecocardiograma de 12 derivações de três novas TVs induzíveis a partir de um novo estudo de eletrofisiologia. A morfologia das três TVs não é nada semelhante, sugerindo locais de saída substancialmente diferentes da TV através da cicatriz. No entanto, combinando a anatomia e a fisiologia, os autores escolheram de forma inteligente uma área de cicatriz que abrangesse todos os três circuitos de TV, e a supressão da TV foi o resultado para esse paciente.

Mas por que a TV não desapareceu imediatamente? Por que o paciente ainda teve algumas TVs nos meses seguintes? Ainda temos muito a aprender sobre os efeitos da radiação no miocárdio cicatrizado e saudável, mas temos décadas de experiência no tratamento de tumores com SBRT. Quando os tumores são tratados com SBRT, muitas vezes leva semanas antes que eles encolham e desapareçam. O efeito biológico da destruição não é imediato e, se a “ablação” cardíaca é o que esperamos com 25 Gy de radiação, podemos esperar uma escala de tempo semelhante. Embora existam claras vantagens de a radioablação ser rápida, não invasiva e de espessura total, a desvantagem da radioablação pode ser ter que esperar por um efeito ablativo benéfico.

Uma grande preocupação dos radioterapeutas é o efeito tóxico da irradiação intencional de células cardíacas saudáveis. Um conjunto considerável de evidências implica, direta ou indiretamente, que a exposição à radiação cause danos ao coração de maneira dose-dependente. Os radioterapeutas fazem de tudo para evitar a aplicação de radiação nos corações de pacientes com tumores torácicos, como câncer de pulmão, mama e esôfago, por medo de causar danos ao coração, e cardiomiopatia. É interessante notar nesse caso que a fração de ejeção era de 20% antes do tratamento (com sintomas de insuficiência cardíaca classe 2–3 da NYHA) e, um ano depois, a fração de ejeção era de 30% (com sintomas classe 1 da NYHA). É reconfortante que esse paciente não tenha sofrido uma redução drástica na função cardíaca após a radiação intencional.

Para avançar com segurança, o campo da radioablação cardíaca precisará de estudos clínicos prospectivos cuidadosos que atentem para os efeitos positivos e negativos da radiação cardíaca intencional. Como a radiação pode ter efeitos que perdurem por muitos anos após a exposição, o seguimento do paciente deve se estender por muitos anos. Equipes científicas básicas e translacionais dedicadas serão cruciais para ajudar a elucidar os diversos efeitos da radiação nas probabilidades de ocorrência de arritmia e no desempenho do miocárdio.
