# Supplementary material for: Radioablação Cardíaca não Invasiva para Doença de Chagas
Source: Arq Bras Cardiol. 2023 Mar 16;120(2):e20230055. [Article in Portuguese] doi: 10.36660/abc.20230055 (PMC10392856; doi:10.36660/abc.20230055)
Supplement: Translation

# Translation: Noninvasive Cardiac Radioablation for Chagas Heart Disease

**Authors:** Philip C. Wiener, Kaitlin M.S. Moore, Carlos Contreras, Clifford G. Robinson, Phillip S. Cuculich

**Affiliations:** 1 USA Washington University School of Medicine, St. Louis, MO – USA

**Keywords:** Chagas Cardiomyopathy, Chagas Disease, Radiosurgery/methods, Use of Radiation in Medicine Research, Tachycardia Ventricular

Chagas heart disease is a common cause of nonischemic cardiomyopathy in Latin America.^1^ It is caused by Trypanosoma cruzi, a parasite that causes acute myopericarditis and a more chronic fibrosing cardiomyopathy. In most studies, the most common overall cause of death is sudden cardiac arrest from arrhythmias (55–60%), followed by heart failure (25–30%) and embolic events (10–15%). implantable cardioverter-defibrillator (ICD) shocks save lives from ventricular arrhythmias but are painful and associated with accelerated patterns of heart failure and death. Catheter ablation can be an effective stop-gap therapy for many patients to minimize ICD shocks from ventricular tachycardia (VT). Because of the transmural patchy nature of scar associated with Chagas disease, the best results are achieved with mapping and ablating the scar from both the endocardial and epicardial surfaces in an effort to homogenize the scar.^2^ And, yet, for many patients, VT recurs, and with it comes more ICD shocks. Recurrences of VT can be the result of incompletely treated circuits within the scar or new circuits forming over time.

Scanavacca et al.^3^ here report the first use of an entirely noninvasive treatment by means of focused radiation in a patient with Chagas to minimize, then eliminate, VT. This therapy, known as stereotactic body radiation therapy (SBRT), has revolutionized the way that many patients with cancers are treated. SBRT is an ablative radiation modality employing advanced imaging technologies capable of accurately damaging a target tissue with a high dose of radiation, while sparing nearby normal tissues. Cardiac SBRT, or cardiac radioablation, requires close collaboration between heart rhythm, radiation oncology, and medical physics teams to develop precise and safe treatment plans within parts of scarred myocardium while avoiding healthy nearby structures. Recently, this technology has been used to treat patients with recurrent VT with promising results in patients with ischemic and various forms of nonischemic cardiomyopathies.^4,5^

So why Chagas? In many ways, Chagas may be an ideal cardiomyopathy to make use of noninvasive cardiac radioablation. First, the VT circuits in Chagas often traverse across the layers of myocardium, using epicardial, midmyocardial, and endocardial scar channels. An important advantage of cardiac radioablation over catheter treatment is that radioablation is delivered across the full thickness of the scarred myocardium. Second, radioablation is a much easier procedure for patients to tolerate. Catheter procedures for patients with Chagas, using both endocardial and epicardial ablation, can be lengthy and risky. Indeed, the patient described in the case report suffered several known complications of epicardial mapping including hemopericardium and hemoperitoneum. With cardiac radioablation, the treatment was completed in 15 minutes, without any catheters introduced into the body. Third, although ventricular arrhythmias can arise from various locations, the most common topographic scar location is the inferolateral region of the left ventricle. This location is well-suited for cardiac radioablation. Luminal structures of the gastrointestinal system (esophagus, stomach, bowel) are radiosensitive, and rare adverse radiation effects to these organs have been described in patients who underwent cardiac radioablation.^6^

The authors are to be commended for several careful and thoughtful aspects of this case. First, they decided on an ideal patient for cardiac radioablation, with more symptoms related to VT than to heart failure. For patients with NYHA class 4 heart failure symptoms, successfully treating VT with catheters or radiation is unlikely to impact the short remaining duration of life. Moreover, patients with advanced cardiomyopathy and extreme levels of myocardial scarring are likely to have many, many ventricular arrhythmias which are not likely to be fully treated with a single procedure. In this case report, however, the authors were thoughtful in their patient selection, choosing a patient with mild-moderate heart failure symptoms and who had already tried conventional therapies. If his VT was controlled with radioablation, it was expected that he would continue along a reasonable life trajectory. Fortunately, that was his experience after radioablation.

Second, from a technical and cognitive perspective, the most challenging part of cardiac radioablation for an electrophysiologist is deciding on the location to irradiate. This process, called “targeting,” requires advanced levels of knowledge of cardiac anatomy and interpretation of electrophysiology data, such as previous catheter maps and 12-lead ECG during VT. The authors made use of several imaging modalities to locate the scar, including previous catheter voltage maps and cardiac CT. Then, they carefully added electrophysiologic data from previous catheter activation mapping and 12-lead ECGs of three new inducible VTs from a new electrophysiology study. The morphology of the three VTs are not at all similar, suggesting substantially different exit sites of VT through the scar. However, by combining the anatomy and physiology, the authors intelligently chose an area of scar which encompass all three VT circuits, and VT suppression was the result for this patient.

But why did VT not disappear immediately? Why did the patient still have a few VTs in the months to follow? We still have much to learn about the effects of radiation on scarred and healthy myocardium, but we do have decades of experience of treating tumors with SBRT. When tumors are treated with SBRT, it often takes weeks before they shrink and disappear. The biologic effect of destruction is not immediate, and if cardiac “ablation” is what we expect with 25 Gy of radiation, then we may expect a similar time scale. While there are clear advantages of radioablation being fast, noninvasive, and full thickness, the disadvantage of radioablation may be having to wait for a beneficial ablative effect.

A major concern of radiation oncologists is the toxic effect of intentional irradiation of healthy heart cells. A considerable body of evidence directly or indirectly implicates radiation exposure as causing harm to the heart in a dose-dependent fashion. Radiation oncologists go to great lengths to avoid delivering radiation to hearts of patients with thoracic tumors like lung, breast, and esophageal cancers for fear of causing heart damage and cardiomyopathy. It is interesting to note in this case that the ejection fraction was 20% before treatment (with NYHA class 2–3 heart failure symptoms), and one year later had an ejection fraction of 30% (with NYHA class 1 symptoms). It is reassuring that this patient did not suffer a dramatic reduction in heart function after intentional radiation.

To safely move forward, the field of cardiac radioablation will need careful prospective clinical trials which remain vigilant for finding positive and negative effects of intentional cardiac radiation. Because radiation can have lasting effects many years after exposure, patient follow-up should extend for many years. Dedicated basic and translational science teams will be crucial to help elucidate the various effects of radiation on both arrhythmia probabilities and myocardial performance.
